# Subjective Cognitive Decline‐Plus: Longitudinal Assessment and Risk for Cognitive Impairment

**DOI:** 10.1002/alz.088004

**Published:** 2025-01-03

**Authors:** Moonil Kang, Clara Li, Arnav Mahajan, Jessica Spat‐Lemus, Shruti Durape, Jiachen Chen, Ashita S. Gurnani, Sherral A. Devine, Sanford H. Auerbach, Ting Fang Alvin Ang, Richard Sherva, Wei Qiao Qiu, Kathryn L. Lunetta, Rhoda Au, Lindsay A. Farrer, Jesse Mez

**Affiliations:** ^1^ Framingham Heart Study, Boston University Chobanian & Avedisian School of Medicine, Boston, MA USA; ^2^ Department of Medicine (Biomedical Genetics), Boston University Chobanian & Avedisian School of Medicine, Boston, MA USA; ^3^ Department of Psychiatry, Icahn School of Medicine at Mount Sinai, New York, NY USA; ^4^ George Washington University School of Medicine and Health Sciences, Washington, DC USA; ^5^ Department of Psychology, Montclair State University, Montclair, NJ USA; ^6^ Department of Neurology, Boston University Chobanian & Avedisian School of Medicine, Boston, MA USA; ^7^ Alzheimer’s Disease Research Center, Boston University Chobanian & Avedisian School of Medicine, Boston, MA USA; ^8^ Department of Biostatistics, Boston University School of Public Health, Boston, MA USA; ^9^ Boston University Chobanian & Avedisian School of Medicine, Boston, MA USA; ^10^ Department of Anatomy & Neurobiology, Boston University Chobanian & Avedisian School of Medicine, Boston, MA USA; ^11^ Slone Epidemiology Center, Boston University Chobanian & Avedisian School of Medicine, Boston, MA USA; ^12^ Department of Pharmacology, Physiology & Biophysics, Boston University Chobanian & Avedisian School of Medicine, Boston, MA USA; ^13^ Department of Psychiatry, Boston University Chobanian & Avedisian School of Medicine, Boston, MA USA; ^14^ Department of Epidemiology, Boston University School of Public Health, Boston, MA USA; ^15^ Department of Ophthalmology, Boston University Chobanian & Avedisian School of Medicine, Boston, MA USA; ^16^ Boston University Alzheimer’s Disease Research Center, Boston, MA USA; ^17^ Framingham Heart Study, Boston, MA USA

## Abstract

**Background:**

Subjective cognitive decline (SCD) is recognized to be in the Alzheimer’s disease (AD) cognitive continuum. An international working group known as the SCD‐initiative recently proposed “SCD plus” features, including report of memory problems, recent SCD relative to conversion, SCD over age 60, and consistent SCD over time, that increase the risk for future objective cognitive decline. These have not been fully assessed in a large community‐based setting.

**Method:**

Participants from all cohorts of the Framingham Heart Study who were ≥ 60 years with normal cognition at analytic baseline were included. SCD was assessed longitudinally using a single question. We treated SCD as a time‐varying variable and considered SCD to be present if it was endorsed at the last cognitively normal visit. If SCD was endorsed during two or more consecutive visits, including the last valid visit, it was considered to begin at the first of the consecutive visits. Outcomes included consensus‐diagnosed mild cognitive impairment (MCI), AD, and all‐cause dementia. Survival analyses were performed on time‐to‐event data obtained between 2005 and 2019 with up to 12 years of follow‐up. Models were adjusted for baseline age, sex, education, *APOE ε*4 status, and tertiles of AD polygenic risk score (PRS) excluding the *APOE* region.

**Result:**

3,735 participants (mean baseline age: 68.0 years, women: 55.1%, non‐Hispanic Whites: 91.6%, SCD: 44.5%, *APOE* *ε*4 carriers: 21.5%) were included. On average, SCD preceded MCI by 4.4 years, AD by 6.8 years, and all‐cause dementia by 6.9 years. *APOE* *ε*4 and tertiles of AD PRS status did not significantly differ between SCD and non‐SCD groups. SCD was significantly associated with survival time to MCI (HR = 1.57; 95% CI = 1.22−2.03; *P* = 4.79×10^−4^), AD (HR = 2.98; 95% CI = 1.89−4.70; *P* = 2.80×10^−6^), and all‐cause dementia (HR = 2.14; 95% CI = 1.44−3.18; *P* = 1.82×10^−4^). After adjustment for *APOE* and AD PRS, the effects of SCD were largely unchanged.

**Conclusion:**

In a community setting, SCD reflecting “SCD plus” features increased the risk of future MCI, AD, and all‐cause dementia with about the same HR estimated in clinic‐based settings. SCD may be an independent clinically expressed risk factor for preclinical AD and other dementias beyond the risk incurred by *APOE* *ε*4 and AD PRS.

